# Cumulative Lifetime Trauma Exposure Among Employed Mental Health Care Professionals and Associations With Professional Quality of Life: A Cross‐Sectional Survey

**DOI:** 10.1111/jpm.70192

**Published:** 2026-08-10

**Authors:** Jacob Hvidhjelm, Lene Lauge Berring

**Affiliations:** ^1^ Department of Research Central and West Zealand Hospital, Region Zealand Slagelse Denmark; ^2^ Department of Forensic Psychiatry Central and West Zealand Hospital, Region Zealand Slagelse Denmark; ^3^ Department of Regional Health Research University of Southern Denmark Odense Denmark; ^4^ University College Absalon Roskilde Denmark

**Keywords:** burnout, compassion fatigue, life change events, mental health personnel, professional, psychiatric nursing, secondary, stress disorders, traumatic, trauma‐informed care

## Abstract

**Introduction:**

Mental health care professionals face emotionally demanding work and often carry invisible personal trauma histories.

**Aim:**

To examine cumulative lifetime trauma exposure among employed psychiatric mental health care professionals and its associations with burnout, secondary traumatic stress, and compassion satisfaction.

**Method:**

A cross‐sectional survey among 309 staff (31.4% registered nurses, 43.7% healthcare assistants, 12.0% psychiatrists and psychologists, 12.9% other) at a Danish psychiatric centre assessed cumulative lifetime trauma exposure, operationalised as a count of ten potentially traumatic event categories, using the Brief Trauma Questionnaire and professional quality of life (ProQOL).

**Results:**

Among those reporting trauma, 34.3% endorsed two or more categories. Higher trauma exposure was associated with secondary traumatic stress (*β* = 1.27, *p* = 0.005) and burnout (*β* = 1.05, *p* = 0.028) but not compassion satisfaction (*p* = 0.759).

**Discussion:**

Cumulative trauma exposure is a largely invisible background characteristic contributing independently to trauma‐related professional strain. Thisis particularly relevant for nursing, as nurses and nursing support staff constitute 75.1% of the inpatient workforce.

**Limitations:**

Cross‐sectional design and single‐centre sample limit causal inference and generalisability.

**Implications:**

Findings support universal, trauma‐informed organisational approaches that do not require disclosure of personal trauma histories, making them feasible for all staff.

**Recommendations:**

Longitudinal studies should examine associations between trauma exposure and occupational demands.

**Relevance Statement:**

Mental health nursing takes place in emotionally demanding environments where staff are routinely exposed to patients' traumatic experiences. This study demonstrates that employed psychiatric mental health care professionals commonly carry cumulative lifetime trauma histories that are associated with secondary traumatic stress and burnout, independent of workplace exposures. These findings are directly relevant to mental health nursing practice, as they highlight a largely invisible source of workforce vulnerability that is unlikely to be effectively addressed through individual screening or targeted interventions. The findings support the implementation of universal, trauma‐informed organisational strategies that protect all mental health care staff, including nursing staff, regardless of personal trauma history.

## Accessible Summary

**What Is Known on the Subject:**

- Mental health care professionals work in emotionally demanding environments and are at risk of burnout and stress‐related difficulties.
- A substantial proportion of healthcare staff carry personal trauma histories from their lives outside work, which may affect how they respond to the demands of care work.
- Organisations typically focus on workplace incidents as the primary source of staff vulnerability, without considering life course trauma exposure.

**What the Paper Adds to Existing Knowledge:**

- Using a census sampling approach with two validated instruments and multivariable regression models, this study shows that cumulative lifetime trauma exposure is common among employed psychiatric mental health care professionals, with nearly two‐thirds reporting at least one traumatic life event.
- Lifetime trauma exposure was associated with secondary traumatic stress and burnout, but not with compassion satisfaction, suggesting that personal trauma history selectively affects stress‐related dimensions of professional well‐being independently of workplace exposures.

**What Are the Implications for Practice:**

- Because personal trauma histories are broadly distributed across the workforce and do not follow predictable demographic patterns, universal organisational approaches are more appropriate than targeted strategies based on individual characteristics.
- Organisations should prioritise universal, trauma‐informed approaches that create psychologically safe and supportive workplaces for all staff, regardless of personal history.
- This is particularly relevant for mental health nursing, as nurses and nursing support staff make up most of the inpatient workforce and are most directly exposed to patients' traumatic experiences.

## 1 Introduction

Mental health care professionals work in emotionally demanding environments characterised by high stress, complex interpersonal dynamics, and sustained exposure to potentially distressing experiences. These occupational demands place staff at increased risk of psychological strain, including burnout, secondary traumatic stress, and emotional exhaustion (Győri et al. 2024; Morse et al. 2012). Psychiatric mental health nurses are particularly affected, constituting the largest professional group in inpatient settings and facing sustained exposure to patient suffering, workplace violence, and emotionally demanding interactions. Studies consistently report moderate to high levels of burnout and secondary traumatic stress among psychiatric nurses internationally, with comparatively low compassion satisfaction (Batran et al. 2025; Fiskeaux et al. 2025; Lobo et al. 2024; Marshman et al. 2022). While workplace‐related adversity is widely recognised as a central occupational hazard in psychiatric care, trauma exposure among professionals does not originate solely from the work context.

## 2 Background

Cumulative trauma exposure, defined as the accumulation of multiple distinct traumatic event types across the lifespan, is associated with a dose–response increase in psychological distress, even among individuals who do not develop clinical PTSD. This cumulative burden may be particularly relevant in emotionally demanding occupational contexts. A growing body of research suggests that a substantial proportion of mental health professionals enter the workforce with significant personal trauma histories, including adverse childhood experiences and other potentially traumatic life events (Mercer et al. 2023; Williamson et al. 2025). From a life‐course perspective, such experiences may shape stress sensitivity, emotion regulation, and coping capacity, thereby influencing how individuals respond to later occupational demands. Personal trauma exposure may therefore represent an under‐recognised background vulnerability within the mental health workforce.

Epidemiological studies have consistently demonstrated that early‐life adversity is common and associated with adverse mental and physical health outcomes across adulthood (Felitti et al. 1998; Hughes et al. 2017). A meta‐analysis further confirms a high global prevalence of adverse childhood experiences and a dose–response relationship between cumulative adversity and later psychological distress (Madigan et al. 2023; Sangalang and Vang 2017). These findings highlight the relevance of cumulative trauma exposure, rather than isolated events, as a determinant of long‐term vulnerability in emotionally demanding contexts.

Within mental health services, the potential significance of personal trauma histories is particularly salient. Care work often involves sustained emotional labour, repeated exposure to suffering, and frequent engagement with patients' traumatic experiences. A systematic review reported lifetime personal trauma exposure among 19% to 81% of mental health professionals, with most studies demonstrating higher levels of secondary traumatic stress among those with such histories (Henderson et al. 2025). This wide range reflects heterogeneity in trauma definitions, measurement approaches, and professional subgroups across studies. Among psychiatric nurses specifically, personal trauma histories may compound occupational vulnerability, as nurses are simultaneously the professional group most directly exposed to patients' traumatic experiences and the group at greatest risk of work‐related distress (Lobo et al. 2024; Marshman et al. 2022). Potential mechanisms include heightened emotional reactivity to patient trauma narratives, increased stress sensitivity, or reduced emotion regulation capacity, though these were not tested in the present study. Cumulative trauma exposure may therefore contribute meaningfully to compassion fatigue and related outcomes in psychiatric care settings.

Personal and occupational trauma exposures may also interact and reinforce one another. This interplay became especially visible during the COVID‐19 pandemic, when exceptional demands were placed on healthcare systems and staff. Meta‐analyses documented elevated rates of depression, anxiety, and sleep disturbances among healthcare workers, illustrating how sustained occupational stressors can overwhelm even well‐functioning systems (Greenberg et al. 2020; Pappa et al. 2020). Similar patterns have been observed in other crisis contexts, where prolonged exposure to extreme demands has been associated with substantial psychological strain among healthcare professionals (Aljadba et al. 2025; Aqtam 2025). In such settings, pre‐existing trauma exposure may amplify stress responses and contribute to cumulative psychological burden.

Despite growing recognition of both personal and occupational trauma exposures, important gaps remain in the literature. Less is known about how cumulative lifetime trauma exposure is distributed across staff groups within inpatient psychiatric services and how such burden relates to key dimensions of professional quality of life when accounting for workplace‐related risks such as violence and threats. Addressing these gaps is important because if cumulative trauma is broadly distributed and independently associated with professional strain, universal organisational approaches may be more appropriate than targeted interventions based on observable characteristics.

At the organisational level, there is increasing recognition that staff well‐being is shaped not only by individual resilience but also by structural and cultural conditions. Organisational approaches informed by trauma‐informed care principles have been associated with improvements in staff well‐being, particularly when supported by leadership engagement, workforce development, and coherent policy frameworks (Berring et al. 2024; Goldstein et al. 2024; Worawichayawongsa et al. 2025). Developing effective workforce interventions therefore requires attention to both cumulative life‐course vulnerability and the contemporary workplace context in which care is delivered. Universal, trauma‐informed approaches emphasise psychological safety, supportive leadership, predictability, and equitable access to resources for all staff, without requiring individual disclosure of personal trauma histories (Gordon 1983; Lewis et al. 2023; Substance Abuse and Mental Health Services Administration 2014).

## 3 The Study

### 3.1 Aim

The primary aim of this study is to examine the prevalence and distribution of cumulative lifetime trauma exposure, operationalised as exposure to potentially traumatic event categories, among employed mental health care professionals, and to identify demographic, educational, and workplace‐related correlates of cumulative trauma burden. A secondary aim is to assess whether greater cumulative trauma exposure is associated with professional quality of life, operationalised as burnout, secondary traumatic stress, and compassion satisfaction. The study further seeks to examine how cumulative life‐course trauma burden is distributed across staff characteristics, and to consider what this distribution implies for organisational approaches to workforce well‐being and resilience. Based on prior research, we expected that greater cumulative lifetime trauma exposure would be associated with higher secondary traumatic stress and burnout, but not with compassion satisfaction, after adjustment for workplace‐related factors.

## 4 Methods

This study employed a cross‐sectional survey design using anonymised, self‐administered questionnaire data collected from mental health care professionals. The survey assessed lifetime personal trauma exposure, workplace‐related factors, and professional quality of life. Lifetime trauma exposure was measured using the Brief Trauma Questionnaire (BTQ) (Schnurr et al. 1999), and professional quality of life was assessed using the Professional Quality of Life Scale (ProQOL, version 5) (Stamm 2010).

Trauma exposure was conceptualised across two domains: lifetime personal trauma occurring outside the workplace and occupational exposure in the form of patient‐related violence (physical assault) and threats (verbal abuse or intimidation). Demographic and job‐related characteristics, including age, sex, and educational background, were examined as potential correlates of cumulative trauma burden.

Participants were recruited from a single psychiatric centre in Denmark, comprising two specialised inpatient units: a forensic psychiatry unit and a dual diagnosis unit. Recruitment was conducted through their employing organisations. Staff were eligible if they were currently employed at one of the two participating units. No exclusion criteria were applied based on age, professional role, or trauma history. The study population comprised frontline mental health professionals across disciplines, including psychiatrists, psychologists, psychiatric nurses, health care assistants, and staff with other educational background (e.g., Physiotherapist and Occupational therapist). All participants provided informed consent prior to participation. Ethical approval was obtained from the relevant institutional review body, and confidentiality was ensured throughout data collection and analysis. Of the 445 invited staff, 309 participated, yielding a response rate of 69.4%. Non‐respondents were part of the same staff population; no further data were available on those who did not participate. Data were collected between October 2023 and February 2024. Data collection spanned 5 months. No major organisational changes or critical incidents were reported at the participating units during this period. As all eligible staff at the participating units were invited to participate, no formal sample size calculation was performed; the achieved sample size was determined by the size of the available population.

Lifetime trauma exposure was assessed using the Brief Trauma Questionnaire, which captures exposure to ten categories of potentially traumatic events across the lifespan. A cumulative trauma score (BTQ Total Trauma) was calculated for each participant by summing the number of endorsed trauma types, yielding a total score ranging from 0 to 10. The BTQ is derived from the Brief Trauma Interview and has demonstrated good test–retest reliability and construct validity, with scores correlating well with interview‐based measures of trauma exposure (Schnurr et al. 2002). The BTQ was administered in a Danish translation prepared for this study, which has not been formally validated. Consistent with its intended purpose, the BTQ was used in the present study to operationalise cumulative lifetime trauma exposure as a count of distinct event categories rather than as a clinical outcome measure, reflecting exposure history rather than trauma‐related symptomatology or psychopathology.

Occupational exposure was assessed using two single self‐report items with a 12‐month reference period. Participants were asked how often they had been exposed to physical violence at their workplace and how often they had been exposed to threats of violence at their workplace. Both items used the response options never, rarely, monthly, weekly, and daily. These variables were included as explanatory factors in analyses of cumulative trauma burden and professional quality of life.

Professional quality of life was assessed using ProQOL Version 5, which measures compassion satisfaction, burnout, and secondary traumatic stress (Stamm 2010). Higher scores on compassion satisfaction reflect more positive professional functioning, whereas higher burnout and secondary traumatic stress scores indicate greater occupational strain. The ProQOL has been validated across various populations and languages, demonstrating acceptable internal consistency. Typically, Cronbach's alpha ranges from 0.85 to 0.90 for compassion satisfaction, 0.80 to 0.82 for secondary traumatic stress, and 0.69 to 0.75 for burnout in international samples (Lenz et al. 2024). In the present sample, Cronbach's alpha was 0.87 for compassion satisfaction, 0.69 for burnout, and 0.76 for secondary traumatic stress. The lower value for burnout is consistent with the pattern reported across international validation studies. Convergent validity has been established through moderate to strong correlations with related measures of occupational distress and well‐being, while discriminant validity has been demonstrated against constructs unrelated to work (Geoffrion et al. 2019; Singh et al. 2024). Construct validity has been assessed using confirmatory factor analysis across multiple samples; fit indices have generally been suboptimal for the original three‐factor model, but bifactor modelling suggests that the three subscales can still be used as distinct dimensions of professional quality of life (Geoffrion et al. 2019; Hotchkiss and Wong 2022). Given the sample size (*n* = 309), confirmatory factor analysis of the ProQOL was not performed, and the three subscales were used as specified by the instrument developer. The substantial conceptual and statistical overlap between the burnout and secondary traumatic stress subscales is recognised as a psychometric limitation and is discussed in the limitations section of this study.

### 4.1 Statistical Analysis

Analyses were conducted using R (version 4.5.2). No formal sample size calculation was performed. The study sample comprised all eligible staff employed at the participating units during the data collection period, and the achieved sample size was therefore determined by the size of the available population. Consequently, associations that are not statistically significant should be interpreted with caution, as the study may have been underpowered to detect small effect sizes. Descriptive statistics were used to summarise participant characteristics and the distribution of cumulative lifetime trauma exposure. Continuous variables are presented as means and standard deviations or medians and interquartile ranges, as appropriate, while categorical variables are presented as frequencies and percentages.

Cumulative lifetime trauma exposure was operationalised using the BTQ Total Trauma score. Its distribution was examined using summary statistics and graphical inspection, and prevalence estimates were calculated for having experienced at least one trauma and for higher trauma thresholds to describe accumulation of trauma exposure within the sample.

To identify demographic, educational, and workplace‐related correlates of cumulative trauma burden, Poisson regression models were fitted with BTQ Total Trauma as the outcome variable, reflecting its nature as count data. Sex, age, educational background, and exposure to workplace threats and violence were included as covariates based on theoretical relevance. To improve model stability and reduce sparse data issues, workplace threats were categorised into three levels: low (never or rarely), moderate (monthly), and high (weekly or daily), and workplace violence was dichotomised as lower (never) vs. highest (rarely or more frequent) exposure. The dispersion parameter was 1.26, indicating mild overdispersion, and robust standard errors (HC0) were therefore applied. Threats were kept as three categories because monthly exposure (12.3%) gave sufficient cell sizes, whereas violence was dichotomised because monthly exposure was rare (0.6%).

Associations between cumulative lifetime trauma exposure and professional quality of life were examined using separate multivariable linear regression models with burnout, secondary traumatic stress and compassion satisfaction as outcomes, operationalised as *t*‐scores from the ProQOL scale. BTQ Total Trauma was entered as a continuous predictor to assess dose–response associations. All models were adjusted for demographic, educational, and workplace‐related variables, and model assumptions were evaluated using residual diagnostics. Scatterplots indicated approximately linear associations between BTQ Total Trauma and each outcome. Quadratic terms were added in sensitivity analyses and were not significant for any outcome (burnout *p* = 0.921, secondary traumatic stress *p* = 0.538, compassion satisfaction *p* = 0.990).

No missing data occurred on the model variables (*n* = 309). Non‐response was examined by comparing respondents and non‐respondents on department, sex, and professional background, using chi‐square tests. All statistical tests were two‐tailed, with statistical significance set at *p* < 0.05 and 95% confidence intervals (CI) reported throughout. No correction for multiple comparisons was applied to the primary associations in Table 4, which tested prespecified hypotheses. The item‐level analyses in Table S1 used Benjamini–Hochberg correction. Exploratory interaction analyses tested whether the association between BTQ Total Trauma and burnout or secondary traumatic stress differed by sex, age group, or professional role. No interaction was significant (all *p* > 0.10).

### 4.2 Artificial Intelligence

AI (Claude) was used only for language editing and reformulation of text during manuscript preparation. All data analysis, interpretation, and final content were carried out and approved by the authors, who take full responsibility for the accuracy and integrity of the work.

## 5 Results

Of the 445 invited mental health care professionals working in inpatient psychiatric units, 309 participated in the study, yielding an overall response rate of 69.4%. Non‐respondents (*n* = 136) were compared to respondents on the three characteristics available for the full invited sample: department, sex, and professional background. Response differed by unit. The response rate was lower among staff in the dual diagnosis unit (59.4%, 92/155) than in the forensic unit (74.8%, 217/290), and this difference was statistically significant (χ^2^(1) = 10.68, *p* = 0.001). Response did not differ by sex, with comparable rates among men (71.8%, 84/117) and women (68.6%, 225/328; χ^2^(1) = 0.28, *p* = 0.60). Response rates by professional background ranged from 63.8% among psychiatrists and psychologists to 83.8% among staff in the other‐education category, but this difference did not reach conventional levels of statistical significance (χ^2^(3) = 6.79, *p* = 0.08). Non‐response was thus unevenly distributed across units, with staff in the dual diagnosis unit underrepresented among respondents relative to their share of the total sample. Participant characteristics and workplace exposure variables are presented in Table 1. The sample was predominantly female (72.8%), with the largest age groups being 41–50 years and 51–60 years (each 29.8%). The mean age, estimated from category midpoints, was 47 years (SD = 12.1). Most participants were mental health nursing staff, comprising registered nurses (31.4%) and health care assistants (43.7%), together accounting for 75.1% of the sample. Psychiatrists and psychologists together comprised 12.0%.

**TABLE 1:** Participant characteristics and workplace exposure (*N* = 309).

Characteristic	*n* (%)
**Sex**	
Female	225 (72.8)
Male	84 (27.2)
**Age**	
20–30 years	38 (12.3)
31–40 years	43 (13.9)
41–50 years	92 (29.8)
51–60 years	92 (29.8)
61–70 years	44 (14.2)
**Education**	
Registered nurses (RNs)	97 (31.4)
Other educational background	40 (12.9)
Psychiatrists and psychologists	37 (12.0)
Social‐ and Healthcare Assistants	135 (43.7)
**Workplace threats**	
Never	112 (36.2)
Rarely	139 (45.0)
Monthly	38 (12.3)
Weekly	19 (6.1)
Daily	1 (0.3)
**Workplace violence**	
Never	243 (78.6)
Rarely	64 (20.7)
Monthly	2 (0.6)

*Note:* Values are number (percentage). Workplace threats refer to threats of violence, and workplace violence refers to physical violence, both from patients and within the past 12 months.

Exposure to workplace threats was common, with 63.8% of participants reporting at least occasional threats and 18.7% reporting monthly, weekly, or daily exposure. Workplace violence was less frequent, with 21.3% reporting rare or monthly exposure and the majority reporting no exposure (78.6%). Monthly workplace violence was reported by only two participants (0.6%).

The distribution of cumulative lifetime trauma exposure, measured using the BTQ Total Trauma score, is shown in Table 2. The mean BTQ score was 1.23 (SD = 1.27), with a median of 1 (IQR = 2) and an observed range of 0–6. Overall, 36.2% of participants reported no lifetime trauma exposure, whereas 63.8% reported exposure to at least one lifetime traumatic event. Among those reporting trauma exposure, 34.3% endorsed two or more trauma types. Higher levels of cumulative trauma exposure were less common, with 16.8% reporting three or more trauma types and 1.6% reporting five or more.

**TABLE 2:** Prevalence and distribution of cumulative lifetime trauma exposure (BTQ Total Trauma).

Measure	Value
Mean (SD)	1.23 (1.27)
Median (IQR)	1 (2)
Range (observed)	0–6
BTQ = 0	112 (36.2%)
BTQ ≥ 1	197 (63.8%)
BTQ ≥ 2	106 (34.3%)
BTQ ≥ 3	52 (16.8%)
BTQ ≥ 5	5 (1.6%)

*Note:* BTQ represents the cumulative number of experienced trauma types across the lifespan.

Abbreviation: BTQ, Brief Trauma Questionnaire.

Associations between demographic, educational, and workplace‐related factors and cumulative lifetime trauma exposure are presented in Table 3. In Poisson regression models adjusted for all covariates, most demographic and educational variables were not significantly associated with BTQ Total Trauma scores. No statistically significant associations were observed for sex or age. With respect to educational background, psychiatrists and psychologists had a lower cumulative trauma burden compared with registered nurses (RNs) (IRR = 0.61, 95% CI: 0.39 to 0.97, *p* = 0.036), while no significant differences were observed for other educational groups. In unadjusted analysis, the estimate for psychiatrists and psychologists was similar (IRR = 0.63, 95% CI: 0.42 to 0.93, *p* = 0.043).

**TABLE 3:** Demographic, educational, and workplace correlates of cumulative lifetime trauma exposure.

Predictor	IRR	95% CI	*p*
Sex (female vs. male)	0.81	0.63–1.04	0.096
Age (per category increase)	0.93	0.84–1.02	0.142
Social‐ and healthcare assistants vs. registered nurses (RNs)	1.11	0.85–1.45	0.451
Psychiatrists and psychologists vs. registered nurses (RNs)	0.61	0.39–0.97	0.036
Other educational background vs. registered nurses (RNs)	0.69	0.44–1.07	0.098
Workplace threats – Moderate vs. low	0.97	0.72–1.32	0.860
Workplace threats – High vs. low	1.10	0.74–1.64	0.641
Workplace violence – Highest vs. lower	1.27	0.99–1.64	0.064

*Note:* Outcome variable was cumulative lifetime trauma exposure (BTQ Total Trauma, range 0–10). Poisson regression models with HC0 robust standard errors were fitted. Workplace threats were categorised as low (never or rarely), moderate (monthly), or high (weekly or daily), and workplace violence was dichotomised as lower (never) vs. highest (rarely or more frequent) exposure. All models were adjusted for all variables shown.

Abbreviation: IRR, incidence rate ratio.

With respect to workplace‐related factors, neither workplace threat exposure nor workplace violence was significantly associated with cumulative lifetime trauma burden after adjustment for all covariates. The estimate for workplace violence was not statistically significant (IRR = 1.27, 95% CI: 0.99 to 1.64, *p* = 0.064).

Associations between cumulative lifetime trauma exposure and professional quality of life outcomes are shown in Table 4. Higher BTQ Total Trauma scores were associated with higher levels of burnout (*β* = 1.05, 95% CI: 0.17 to 1.94, *p* = 0.028) and secondary traumatic stress (*β* = 1.27, 95% CI: 0.39 to 2.16, *p* = 0.005), independent of demographic, educational, and workplace‐related factors. No significant association was observed between cumulative trauma exposure and compassion satisfaction (*β* = −0.14, 95% CI: −1.04 to 0.76, *p* = 0.759). Standardised coefficients for BTQ Total Trauma were 0.07 for burnout and 0.14 for secondary traumatic stress. Psychiatrists and psychologists reported higher compassion satisfaction than registered nurses (mean difference = 4.87 *t*‐score points, 95% CI: 0.93 to 8.81, *p* = 0.016), though no other educational differences were observed for this outcome.

**TABLE 4:** Associations between cumulative lifetime trauma exposure and professional quality of life.

Predictor	Burnout *β* (95% CI)	*p*	Secondary traumatic stress *β* (95% CI)	*p*	Compassion satisfaction *β* (95% CI)	*p*
BTQ Total Trauma (per trauma)	1.05 (0.17 to 1.94)	0.028	1.27 (0.39 to 2.16)	0.005	−0.14 (−1.04 to 0.76)	0.759
Sex (female vs. male)	−1.25 (−3.81 to 1.30)	0.335	−0.08 (−2.64 to 2.48)	0.953	1.77 (−0.83 to 4.36)	0.181
Age (per category increase)	0.45 (−0.52 to 1.41)	0.367	0.49 (−0.49 to 1.46)	0.327	0.26 (−0.72 to 1.24)	0.604
Health care assistant's vs. registered nurses (RNs)	1.21 (−1.44 to 3.86)	0.369	−0.04 (−2.69 to 2.61)	0.975	−1.03 (−3.72 to 1.65)	0.450
Psychiatrists and psychologists vs. registered nurses (RNs)	−2.27 (−6.16 to 1.62)	0.252	−0.54 (−4.43 to 3.35)	0.785	4.87 (0.93 to 8.81)	0.016
Other educational background vs. registered nurses (RNs)	−0.42 (−4.08 to 3.24)	0.821	1.79 (−1.88 to 5.45)	0.338	4.25 (0.54 to 7.97)	0.025
Workplace threats – Moderate vs. low	3.68 (0.26 to 7.10)	0.035	1.93 (−1.49 to 5.35)	0.269	−2.22 (−5.68 to 1.25)	0.209
Workplace threats – High vs. low	6.81 (2.24 to 11.4)	0.004	7.71 (3.13 to 12.3)	0.001	−1.06 (−5.70 to 3.57)	0.653
Workplace violence – Highest vs. lower	−0.75 (−3.57 to 2.07)	0.600	1.32 (−1.51 to 4.14)	0.360	1.44 (−1.42 to 4.29)	0.324

*Note: β* coefficients represent adjusted mean differences in outcome *t*‐scores per unit increase in the predictor. Outcomes were burnout, secondary traumatic stress, and compassion satisfaction *t*‐scores from the Professional Quality of Life Scale (ProQOL version 5). Workplace threats were categorised as low (never or rarely), moderate (monthly), or high (weekly or daily), and workplace violence was dichotomised as lower (never) vs. highest (rarely or more frequent) exposure. All models were adjusted for sex, age, educational background, and workplace‐related variables.

In adjusted models, higher workplace threat exposure was associated with higher burnout and secondary traumatic stress scores. Compared with low exposure, high levels of workplace threats were associated with higher burnout (*β* = 6.81, 95% CI: 2.24 to 11.4, *p* = 0.004) and higher secondary traumatic stress (*β* = 7.71, 95% CI: 3.13 to 12.3, *p* = 0.001). A comparison of these two coefficients did not show a significant difference (*z* = 0.71, *p* = 0.476). Moderate threat exposure was significantly associated with burnout (*β* = 3.68, 95% CI: 0.26 to 7.10, *p* = 0.035) but not with secondary traumatic stress (*β* = 1.93, 95% CI: −1.49 to 5.35, *p* = 0.269). Workplace violence was not significantly associated with either outcome. No consistent associations were observed for sex, age, or educational background in these models.

Exploratory analyses of individual BTQ trauma categories are presented in Table S1. Individual trauma categories varied in prevalence. The most frequently reported were witnessing serious injury or death (Q10, 24.6%), serious accident (Q2, 22.7%), and physical assault (Q6, 22.0%), while sexual assault (Q7) was reported by 4.2%. Two categories, serious accident (Q2) (*β* = 3.90, 95% CI: 1.25 to 6.56, *p* = 0.004, FDR‐*p* = 0.030) and physical assault (Q6) (*β* = 3.79, 95% CI: 1.10 to 6.49, *p* = 0.006, FDR‐*p* = 0.030), remained significantly associated with secondary traumatic stress after correction for multiple testing. Stratified analyses by unit showed that associations between cumulative trauma exposure and secondary traumatic stress and burnout were broadly consistent across the two units, although estimates were attenuated and non‐significant in the smaller dual diagnosis unit (*n* = 92), likely reflecting reduced statistical power. A significant interaction was found for compassion satisfaction (*p* = 0.016). In the forensic unit (*n* = 217) the association between BTQ Total Trauma and compassion satisfaction was negative and non‐significant (*β* = −0.75, *p* = 0.15), whereas in the dual diagnosis unit (*n* = 92) it was positive and close to significance (*β* = 1.73, *p* = 0.07). The interaction thus reflects opposite directions of association across units, though neither estimate reached significance on its own. Exploratory analyses testing whether the associations between BTQ Total Trauma and each ProQOL outcome differed by sex, age, or professional role were largely non‐significant (*p* ≥ 0.10). Two exceptions approached significance: the interaction between BTQ Total Trauma and sex for compassion satisfaction (*p* = 0.064), and between BTQ Total Trauma and age for secondary traumatic stress (*p* = 0.094).

## 6 Discussion

This study examined cumulative lifetime trauma exposure among mental health care professionals working in inpatient psychiatric settings, its distribution across staff characteristics, and its associations with professional quality of life. Lifetime trauma exposure was common, with nearly two‐thirds of participants reporting at least one traumatic event. Notably, most participants were mental health nursing staff, suggesting that cumulative trauma exposure is a widespread background characteristic specifically within the nursing workforce in inpatient psychiatric settings. Cumulative trauma burden was modestly but consistently associated with higher levels of secondary traumatic stress, while associations with burnout were weaker. These associations persisted after adjustment for demographic characteristics, educational background, and workplace‐related exposures, suggesting that cumulative life‐course trauma exposure contributes independently to dimensions of professional strain.

An important interpretive consideration is that lifetime trauma exposure was measured using the BTQ as a count of exposure to categories of potentially traumatic events rather than as an index of trauma‐related symptoms or psychopathology (Schnurr et al. 1999). The BTQ is designed to assess exposure to DSM Criterion A‐type events and does not capture current psychological distress, PTSD diagnosis, subjective trauma impact, or the severity and chronicity of individual events. (Purgato et al. 2023; Schnurr et al. 1999). It also does not assess the individual's subjective emotional response to events, which further distinguishes exposure history from clinical trauma reactions. BTQ Total Trauma therefore reflects cumulative life‐course exposure history, which is conceptually distinct from trauma‐related clinical outcomes, as many trauma‐exposed individuals do not develop persistent psychopathology (Karstoft and Armour 2023; Sumner et al. 2015). The associations observed should thus be understood in terms of exposure history as a background vulnerability factor rather than clinical trauma sequelae. The associations therefore reflect a relation between exposure history, not current PTSD symptoms, and professional quality of life. This suggests that trauma exposure without current clinical symptoms may still be relevant to occupational well‐being.

Cumulative lifetime trauma exposure was common in this sample, but marked variation was evident across individuals. Although most participants reported at least one potentially traumatic event, only a minority endorsed higher cumulative exposure levels. These distributions mirror epidemiological findings indicating that trauma exposure is widespread but unevenly distributed, with relatively few individuals experiencing multiple distinct trauma types. Research further demonstrates dose–response patterns in which increasing numbers of trauma exposures are associated with elevated psychological distress, even though many exposed individuals do not develop persistent clinical symptoms (Benjet et al. 2016; Karstoft and Armour 2023; Madigan et al. 2023). The low proportion of participants with very high trauma counts may therefore contribute to the modest effect sizes observed in associations with professional quality of life. The modest effect sizes may also reflect the BTQ's focus on exposure breadth rather than severity or subjective impact, a measurement characteristic discussed further in the limitations section.

Demographic and educational characteristics showed limited associations with cumulative trauma exposure, and no significant differences were observed across professional groups including registered nurses. Because BTQ reflects life‐course exposures that often predate professional roles and occur outside the workplace context, such vulnerability may remain largely invisible within organisational structures that classify staff by position, seniority, or education. Studies in healthcare and mental health populations similarly suggest that personal trauma histories are not strongly patterned by professional role or sociodemographic variables and cannot be reliably inferred from observable characteristics (Henderson et al. 2025; Lobo et al. 2024). This may help explain the limited subgroup differences observed and suggests that cumulative trauma burden does not align neatly with formal organisational categories. The lower cumulative trauma burden observed among psychiatrists and psychologists may reflect socioeconomic protective factors or selection effects, though this finding warrants cautious interpretation given the small subgroup size. Differential reporting is another possible explanation. Psychiatrists and psychologists may be less likely to disclose personal trauma histories due to professional stigma or concerns about perceived vulnerability, consistent with broader evidence of underreporting in professional groups where disclosure of personal difficulties may carry perceived career or reputational risk (Boskovic et al. 2024; Krumpal 2013).

International and Nordic epidemiological studies confirm that most adults report at least one lifetime trauma exposure, although reported rates vary considerably depending on the instrument and population studied (Benjet et al. 2016; Heir et al. 2019; Kessler et al. 2017), and help contextualise reviews indicating substantial rates of personal trauma histories among mental health professionals (Henderson et al. 2025). General population studies report considerable variation in lifetime trauma exposure, with estimates of 85% to 86% in Norway (Heir et al. 2019), 84.3% in Iceland (Einarsdóttir et al. 2024), and over 70% across 24 countries in the World Mental Health Survey Consortium (Benjet et al. 2016). This variation partly reflects differences in the number and breadth of event categories assessed across instruments (Karstoft and Armour 2023). Using a related but methodologically distinct approach, a large Danish population cohort applying the Cumulative Lifetime Adversity Measure, a 37‐item instrument summing the total number of adverse life events experienced over the life course, including repeated occurrences of the same event type, reported a mean cumulative adversity score of 5.9 (SD 3.7, observed range 0 to 35) (Carstensen et al. 2020). Because this instrument counts recurring events rather than distinct trauma categories, its scores are not directly comparable to BTQ Total Trauma, but its broader item coverage further illustrates how measurement choices shape reported levels of adversity. The prevalence observed in the present study (63.8%) falls toward the lower end of these general population estimates, consistent with the BTQ's more limited set of ten trauma categories. Rather than suggesting that inpatient mental health professionals constitute a uniquely trauma‐exposed group, the present findings indicate that they reflect broader life‐course exposure patterns seen in the general population and among healthcare personnel (Maunder et al. 2010). A substantial proportion therefore enter emotionally demanding roles with prior exposure histories that may remain largely invisible within organisational contexts.

Cumulative lifetime trauma exposure was differentially associated with dimensions of professional quality of life. Greater trauma burden was associated with higher levels of secondary traumatic stress, whereas the association with burnout was weaker and should be interpreted cautiously given its borderline statistical significance. No association was observed with compassion satisfaction. This selective pattern aligns with empirical findings among mental health professionals showing that personal trauma history is more strongly related to secondary traumatic stress than to burnout, which is more closely linked to organisational and workload factors (Henderson et al. 2025; Leung et al. 2023). Within the ProQOL framework, secondary traumatic stress reflects trauma‐related, fear‐based and emotionally resonant responses arising from indirect exposure to others' suffering, whereas burnout is more strongly shaped by structural job demands and perceived inefficacy (Leiter and Maslach 2016; Stamm 2010; West et al. 2018). Cumulative trauma exposure may therefore heighten emotional resonance or stress sensitivity when working with traumatised patients, thereby relating more closely to STS than to the more context‐dependent construct of burnout, and leaving the motivational and meaning‐related dimension of compassion satisfaction largely unaffected (La Mott and Martin 2019; Mercer et al. 2023). Future longitudinal studies should test directly whether emotional resonance, such as empathy or emotional contagion, mediates the relation between personal trauma history and secondary traumatic stress since a longitudinal design would also help establish whether trauma history precedes rather than follows changes in these mechanisms.

The persistence of these associations after adjustment for workplace violence and threats suggests that cumulative trauma exposure reflects a background life‐course vulnerability rather than merely a proxy for current occupational adversity. Studies among mental health professionals indicate that personal trauma histories are common and may increase susceptibility to secondary stress responses without necessarily implying impaired occupational functioning (Henderson et al. 2025; Mercer et al. 2023). At the same time, many professionals with trauma backgrounds report sustained professional engagement and competence, supporting models in which vulnerability is probabilistic rather than deterministic and shaped by contextual and resilience factors (Leung et al. 2023). For example, a recent quantitative study among trainee therapists found that greater childhood physical abuse was positively associated with alliance bond capacity, although the authors caution that this finding requires further investigation and replication (Kästner et al. 2025).

Workplace threat exposure was positively associated with both burnout and secondary traumatic stress, corroborating the broader literature on occupational adversity in psychiatric settings and supporting the role of high‐demand work environments as a contextual contributor to professional strain independent of personal trauma history (Ayed et al. 2024; Henderson et al. 2025; Hensel et al. 2015; Hilton et al. 2022; Rudkjoebing et al. 2020; Tracy and Zadinsky 2025; Wang et al. 2022). Workplace violence was not associated with burnout or secondary traumatic stress. This may reflect its low frequency, as only 21.3% reported any exposure and 0.6% reported monthly exposure, which limits the power to detect an effect.

Together, these findings support a life‐course perspective on workforce well‐being in psychiatric settings. Trauma exposure among staff extends beyond occupational incidents and may constitute an accumulated background characteristic that interacts with the emotional demands of mental health care work, particularly in relation to trauma‐related dimensions of professional strain rather than uniformly impairing professional capacity (Henderson et al. 2025; Park et al. 2025).

### 6.1 Implications for Practice and Organisational Strategies

As cumulative trauma exposure in this study reflects lifetime exposure history rather than current trauma symptoms, these findings should not be interpreted as indicating that staff with trauma histories require identification, categorisation, or clinical labelling. Personal trauma exposure is common among mental health professionals, including psychiatric nurses, and does not in itself imply impaired occupational functioning, as many individuals with such histories maintain effective professional engagement (Henderson et al. 2025; Mercer et al. 2023; Sodeke‐Gregson et al. 2013; Stamm 2010; Yu et al. 2021). Instead, the findings suggest that a proportion of the workforce may carry a pre‐existing life‐course vulnerability that is not visible within routine organisational structures.

Given that cumulative trauma exposure is broadly distributed and shows limited patterning by demographic or professional characteristics, organisational responses that depend on identifying or disclosing personal trauma histories are unlikely to be effective or appropriate. Universal approaches informed by trauma‐informed care principles offer a more suitable framework, emphasising psychological safety, supportive leadership, predictability, and equitable access to resources for all staff. Such approaches address workforce vulnerability at a systems level without requiring individual disclosure or risking the stigmatisation of staff with trauma histories (Berring et al. 2024; Goldstein et al. 2024; Henderson et al. 2025; Mercer et al. 2023; Worawichayawongsa et al. 2025).

The differential associations observed in this study also suggest that distinct mechanisms may underlie secondary traumatic stress and burnout. Interventions addressing STS may benefit from focusing on emotional processing, reflective supervision, structured debriefing, and building psychological safety in the context of exposure to patients' traumatic material, including approaches such as narrative exposure techniques that have shown promise in mental health settings (Webb and Penz 2024). Burnout, by contrast, is more strongly influenced by structural and organisational conditions such as workload, staffing levels, and leadership practices (Leiter and Maslach 2016; Leung et al. 2023; Ruotsalainen et al. 2015; West et al. 2018). Effective workforce strategies may therefore require parallel attention to both trauma‐related and organisational pathways of strain. For mental health nursing specifically, supervision, peer support structures, and trauma‐informed leadership that acknowledge staff vulnerability without requiring individual disclosure are important. Reviews of interventions for psychiatric nurses highlight the importance of multilevel approaches combining psychological support with organisational changes, including improved staffing, visible leadership commitment to safety, and team‐based resilience resources (Marshman et al. 2022; Webb and Penz 2024; Zhang et al. 2025).

Overall, these findings support integrated organisational approaches that acknowledge cumulative life‐course exposure as a background characteristic within the workforce while prioritising system‐level conditions that sustain professional quality of life.

## 7 Methodological Considerations and Limitations

Several limitations should be considered when interpreting these findings. First, the cross‐sectional design precludes causal inference and raises the possibility of reverse causation (Greenland and Robins 1986; Westreich and Greenland 2013). Observed associations may therefore reflect bidirectional relationships or unmeasured confounding rather than temporal effects of trauma exposure on professional quality of life. Current distress, such as secondary traumatic stress, may increase the reporting of past traumatic events, which could inflate the observed associations.

Although the response rate was relatively high for a voluntary occupational survey (69.4%) and compares favourably with response rates commonly reported in surveys of healthcare professionals (Baruch and Holtom 2008; Cook et al. 2000; VanGeest et al. 2007), the possibility of non‐response bias cannot be excluded. Professionals with higher cumulative trauma exposure may have been either more motivated to participate due to personal relevance or less likely to respond due to emotional burden or survey fatigue. Consequently, the observed prevalence of lifetime trauma exposure may represent either an underestimation or overestimation of the true distribution within the workforce.

Reliance on self‐report measures introduces potential recall and social desirability bias, which may lead to underreporting or distortion of trauma exposure and stress responses, particularly in professional groups where stigma or role expectations may influence disclosure (Boskovic et al. 2024; Goniewicz et al. 2024; Krumpal 2013).

Trauma exposure was operationalised using counts of trauma categories on the BTQ (Schnurr et al. 1999). This approach does not capture severity, chronicity, developmental timing, frequency, or subjective meaning of events. Individuals with identical BTQ counts may therefore differ substantially in lived experience and psychological impact. Contemporary methodological discussions highlight that trauma checklists vary considerably in the extent to which they assess intensity, duration, and subjective appraisal, which may constrain interpretability when exposure is reduced to event counts (Karstoft and Armour 2023; Murphy et al. 2015; Shannonhouse et al. 2016). Interpretation is consequently limited to cumulative exposure history rather than trauma burden in a clinical sense, and this measurement characteristic may contribute to modest effect sizes. The study did not assess current PTSD diagnosis or trauma treatment history. We therefore cannot say whether associations differ between staff with active trauma‐related disorders and those with exposure alone. The BTQ also does not record whether participants have received treatment for traumatic events, and prior treatment could weaken the relation between exposure and current professional quality of life. The study did not measure protective factors such as peer support, supervision quality, or coping strategies, so we cannot say whether these modify the relation between trauma exposure and professional quality of life.

Professional quality of life was assessed using the ProQOL, in which burnout and secondary traumatic stress are theoretically distinct constructs but share some variance reflecting general distress (Heritage et al. 2018; Stamm 2010). Empirical studies indicate that these dimensions can show high convergence within compassion fatigue frameworks, at times approaching a shared general distress factor. Empirical psychometric research has noted substantial conceptual and statistical overlap between these dimensions, particularly within compassion fatigue frameworks, which may attenuate observed specificity of associations and complicate interpretation of differential effects (Geoffrion et al. 2019; Heritage et al. 2018). In the present sample, burnout and secondary traumatic stress were moderately correlated (Spearman's rho = 0.47, *p* < 0.001), in line with this overlap. The absence of an association between cumulative trauma exposure and compassion satisfaction should be interpreted cautiously, as null findings cannot confirm the theoretical mechanisms proposed.

Finally, the study was conducted within a single psychiatric centre comprising two specialised inpatient units in Denmark, which limits generalisability, as occupational contexts, health system structures, and cultural norms influence both trauma exposure and professional quality of life. Denmark has a publicly funded health system with strong labour protections, and the findings may not transfer to under‐resourced or privatised mental health systems. Although the findings align with international literature on trauma exposure and professional quality of life among mental health professionals (Batran et al. 2025; Lobo et al. 2024; Vento et al. 2020), caution is warranted when extrapolating to other national or organisational settings (Goniewicz et al. 2024). The lower response rate in the dual diagnosis unit (59.4%) compared with the forensic unit (74.8%) provides an additional reason for cautious interpretation of the stratified findings in that unit. Stratified analyses were broadly consistent across units for burnout and secondary traumatic stress, but the interaction for compassion satisfaction warrants caution. The dual diagnosis unit treats patients with co‐occurring substance use and mental disorders and may differ from the forensic unit in patient acuity and staff support, but the small sample in that unit (*n* = 92) limits interpretation.

## 8 Conclusion

These findings are directly relevant to mental health nursing practice, where nurses constitute the primary workforce in inpatient psychiatric settings and are at the frontline of sustained exposure to patients' traumatic experiences (Fiskeaux et al. 2025; Lobo et al. 2024). Lifetime exposure to potentially traumatic events was common among mental health care professionals working in inpatient psychiatric settings, with nearly two thirds reporting at least one lifetime trauma and more than one third reporting none. Cumulative trauma burden showed limited variation across demographic and educational characteristics. Higher BTQ total scores were associated with higher levels of secondary traumatic stress and, more modestly, burnout after adjustment for sex, age, education, and workplace‐related exposures. Exploratory item‐level analyses identified serious accident (Q2) and physical assault (Q6) as significantly associated with secondary traumatic stress after correction for multiple testing, whereas no item‐level associations with burnout or compassion satisfaction remained significant, supporting the relevance of cumulative exposure alongside these two specific categories. Cumulative trauma exposure was not associated with compassion satisfaction, which suggests that personal trauma history affects stress‐related dimensions of professional well‐being without reducing the satisfaction staff derive from care work. For nurse managers, this means that personal trauma history should not be treated as rare or limited to identifiable subgroups, and that universal support structures fit the findings better than targeted screening.

These findings indicate that lifetime trauma exposure represents a background characteristic within the inpatient psychiatric workforce that may contribute to trauma‐related dimensions of professional strain. Organisational efforts to support staff well‐being should therefore prioritise psychologically safe work environments and resources that do not depend on disclosure or identification of personal trauma histories. Examples include employee assistance programmes, peer support, reflective supervision, and flexible work arrangements available to all staff. Longitudinal studies are needed to establish whether personal trauma exposure precedes and predicts later secondary traumatic stress and burnout, or whether current distress raises the reporting of past events.

## Author Contributions

**Jacob Hvidhjelm:** conceptualization, methodology, formal analysis, writing – original draft, writing – review and editing. **Lene Lauge Berring:** writing – review and editing. All authors approved the final version of the manuscript.

## Funding

The authors have nothing to report.

## Ethics Statement

The study was conducted in accordance with Danish regulations for health research and the principles of the Declaration of Helsinki. The project was reviewed and approved by the Capital Region of Denmark (journal number: 2012‐58‐0004). All participants received written information about the study and provided informed consent prior to participation. Data were collected anonymously and handled in accordance with applicable data protection regulations.

## Conflicts of Interest

The authors declare no conflicts of interest.

## Supporting information


**Table S1:** Exploratory associations between individual lifetime trauma categories (BTQ items) and burnout, secondary traumatic stress, and compassion satisfaction.

## Data Availability

The datasets generated and analysed during the current study are not publicly available due to institutional and data protection regulations but are available from the corresponding author on reasonable request, subject to appropriate approvals.
