# Supplementary material for: Cumulative Lifetime Trauma Exposure Among Employed Mental Health Care Professionals and Associations With Professional Quality of Life: A Cross‐Sectional Survey
Source: J Psychiatr Ment Health Nurs. 2026 Aug 10;33(6):1014–26. doi: 10.1111/jpm.70192 (PMC13748693; doi:10.1111/jpm.70192)
Supplement: Supplementary file 1 [file JPM-33-1014-s001.docx]

**Supplementary**

**Table S1. Exploratory associations between individual lifetime trauma categories (BTQ items) and burnout, secondary traumatic stress, and compassion satisfaction**

| **Outcome** | **BTQ item** | **n exposed (%)** | **Adjusted β (95% CI)** | **p-value** | **p-value (FDR)** |
| --- | --- | --- | --- | --- | --- |
| **Burnout** | BTQ item Q1 | 5 (1.6) | 4.09 (-4.74 to 12.91) | 0.370 | 0.617 |
|  | BTQ item Q2 | 70 (22.7) | 1.82 (-0.85 to 4.49) | 0.181 | 0.463 |
|  | BTQ item Q3 | 17 (5.5) | 2.79 (-2.05 to 7.64) | 0.257 | 0.515 |
|  | BTQ item Q4 | 23 (7.4) | 3.51 (-0.69 to 7.71) | 0.101 | 0.463 |
|  | BTQ item Q5 | 26 (8.4) | -0.49 (-4.52 to 3.54) | 0.811 | 0.968 |
|  | BTQ item Q6 | 68 (22.0) | 2.92 (0.22 to 5.61) | 0.034 | 0.339 |
|  | BTQ item Q7 | 13 (4.2) | 1.14 (-4.41 to 6.69) | 0.686 | 0.858 |
|  | BTQ item Q8 | 20 (6.5) | 3.11 (-1.50 to 7.71) | 0.185 | 0.463 |
|  | BTQ item Q9 | 62 (20.1) | 0.94 (-1.84 to 3.72) | 0.506 | 0.723 |
|  | BTQ item Q10 | 76 (24.6) | 0.22 (-2.38 to 2.82) | 0.865 | 0.994 |
| **Secondary traumatic stress** | BTQ item Q1 | 5 (1.6) | 6.39 (-2.47 to 15.25) | 0.157 | 0.392 |
|  | BTQ item Q2 | 70 (22.7) | 3.90 (1.25 to 6.56) | 0.004 | 0.030 |
|  | BTQ item Q3 | 17 (5.5) | 2.47 (-2.40 to 7.34) | 0.319 | 0.539 |
|  | BTQ item Q4 | 23 (7.4) | 2.13 (-2.11 to 6.36) | 0.323 | 0.539 |
|  | BTQ item Q5 | 26 (8.4) | -0.08 (-4.14 to 3.97) | 0.968 | 0.968 |
|  | BTQ item Q6 | 68 (22.0) | 3.79 (1.10 to 6.49) | 0.006 | 0.030 |
|  | BTQ item Q7 | 13 (4.2) | -0.69 (-6.27 to 4.90) | 0.808 | 0.865 |
|  | BTQ item Q8 | 20 (6.5) | 3.39 (-1.24 to 8.01) | 0.151 | 0.392 |
|  | BTQ item Q9 | 62 (20.1) | 0.72 (-2.08 to 3.52) | 0.613 | 0.876 |
|  | BTQ item Q10 | 76 (24.6) | 0.06 (-2.55 to 2.68) | 0.961 | 0.917 |
| **Compassion satisfaction** | BTQ item Q1 | 5 (1.6) | 5.14 (-3.72 to 14.01) | 0.255 | 0.597 |
|  | BTQ item Q2 | 70 (22.7) | 0.64 (-2.04 to 3.33) | 0.637 | 0.637 |
|  | BTQ item Q3 | 17 (5.5) | 3.47 (-1.39 to 8.33) | 0.161 | 0.536 |
|  | BTQ item Q4 | 23 (7.4) | -1.28 (-5.51 to 2.96) | 0.554 | 0.637 |
|  | BTQ item Q5 | 26 (8.4) | -1.87 (-5.92 to 2.18) | 0.364 | 0.598 |
|  | BTQ item Q6 | 68 (22.0) | -3.38 (-6.08 to -0.68) | 0.014 | 0.144 |
|  | BTQ item Q7 | 13 (4.2) | 4.29 (-1.27 to 9.85) | 0.130 | 0.536 |
|  | BTQ item Q8 | 20 (6.5) | -1.91 (-6.54 to 2.73) | 0.419 | 0.598 |
|  | BTQ item Q9 | 62 (20.1) | -0.68 (-3.48 to 2.11) | 0.631 | 0.637 |
|  | BTQ item Q10 | 76 (24.6) | 1.38 (-1.23 to 3.99) | 0.298 | 0.597 |

**Note:** BTQ items represent individual lifetime trauma categories assessed using the Brief Trauma Questionnaire. Regression coefficients (β) represent adjusted mean differences in ProQOL T-scores (burnout, secondary traumatic stress, or compassion satisfaction) associated with each trauma category (yes vs no). All models were adjusted for sex, age, educational background, workplace threats, and workplace violence. P values were corrected for multiple testing using the Benjamini–Hochberg false discovery rate (FDR) procedure within each outcome. Analyses are exploratory and should be interpreted with caution, particularly for trauma categories with low prevalence.

**Brief Trauma Questionnaire (BTQ):** **Items** **Q1. War zone exposure**: *Have you ever served in a war zone, or have you ever served in a noncombat job that exposed you to war-related casualties (for example, as a medic or on graves registration duty)?* **Q2. Serious accident**: *Have you ever been in a serious car accident, or a serious accident at work or somewhere else?* **Q3. Natural or technological disaster:** *Have you ever been in a major natural or technological disaster, such as a fire, tornado, hurricane, flood, earthquake, or chemical spill?* **Q4. Life-threatening illness:** *Have you ever had a life-threatening illness such as cancer, a heart attack, leukemia, AIDS, multiple sclerosis, etc.?* **Q5. Physical abuse in childhood:** *Before age 18, were you ever physically punished or beaten by a parent, caretaker, or teacher so that you were very frightened, thought you would be injured, or received bruises, cuts, welts, lumps, or other injuries?* **Q6. Physical assault:** *Not including any punishments or beatings already reported, have you ever been attacked, beaten, or mugged by anyone, including friends, family members, or strangers?* **Q7. Sexual assault:** *Has anyone ever made or pressured you into having some type of unwanted sexual contact?* **Q8. Other serious injury or threat to life:** *Have you ever been in any other situation in which you were seriously injured, or feared you might be seriously injured or killed?* **Q9. Violent death of close other:** *Has a close family member or friend died violently, for example, in a serious car crash, mugging, or attack?* **Q10. Witnessing serious injury or death:** *Have you ever witnessed a situation in which someone was seriously injured or killed, or feared someone would be seriously injured or killed? (Excluding events already reported in Items 1–9.)*
